# Movement Coordination’s Link with Common Ground During Dyadic Peer Discourse in Typically Developing and Autistic Speakers

**DOI:** 10.1007/s10803-024-06642-6

**Published:** 2024-11-21

**Authors:** Einat Karin, Ronny Geva, Shahar Bar-Yehuda, Yael Estrugo, Nirit Bauminger-Zviely

**Affiliations:** 1 Faculty of Education, Bar-Ilan University, 52900 Ramat-Gan, Israel; 2 Department of Psychology, The Gonda Brain Research Center, Bar-Ilan University, Ramat-Gan, Israel

**Keywords:** ASD, Common ground, Language-pragmatic, Motor skills, Joint action, Motor coordination

## Abstract

Common ground (CG)—a pragmatic capability that reflects the construction of shared meaning by two interlocutors during conversation—is widely accepted as crucial for effective communication, but its exploration has been limited in the context of children’s peer-to-peer interaction. Specifically, this study aimed to explore CG differences between typically developing (TD) and autistic populations, CG’s developmental trajectories, as well as the link between CG and motor coordination skills during peer interactions. Study participants included 148 children (6–16 years), comparing 64 TD and 84 autistic children across three age levels. Fixed dyads matched on IQ, age, sex, and mother's education performed a CG tangram-card task and complementary joint action (JA) tasks to evaluate peer-to-peer movement coordination. Individual motor skills were also assessed. As expected, both autistic and TD groups showed increasing efficacy over the 6-turn CG task (fewer words and shorter duration to communicate), although autistic participants were less efficient than TD participants. Better motor skills and JA synchronization correlated with both groups’ more efficient CG performance. Additionally, the indirect relationship between group (TD/autism) and CG was mediated by motor skills and JA, with age moderating the relationship between JA and CG. Specifically, better motor skills and socio-motor coordination were associated with more efficient CG creation, particularly in younger children (under 10 years). These findings suggest potential novel avenues for early interventions targeting motor and language challenges in autism to enhance pragmatic abilities and peer interactions, offering insights into language development in this population.

**Supplementary Information:**

The online version contains supplementary material available at 10.1007/s10803-024-06642-6.

## Introduction

When people talk with each other, they move—they may tilt their head towards each other, lean forward, shake their head for “no” or shrug a shoulder to express disinterest or lack of comprehension. This multimodal body-language embodied perception of a conversation requires coordination between the two interlocutors’ words and bodily actions to create an effective interactive process (Mondada, 2016). As such, each speaker’s verbal expression triggers the other’s corresponding motor action and vice versa; thus, language, gesture, gaze, head movements, facial expressions, body posture, and body movements cojoin to create mutual understanding between the interlocutors (Garrod & Pickering, 2009; Pickering & Garrod, 2004). Overall, children create shared knowledge known as “common ground” during a conversation, and they also coordinate their action in time and space to create “joint action” (Okabayashi, 2017). Both common ground (CG) and joint action (JA) seem to be crucial aspects of effective peer interactive conversation, but their cojoined contribution to peer-to-peer communication has been overlooked. Little empirical attention has focused on the mechanisms by which each interlocutor’s own bodily movements and the dyad’s ability to synchronize their motor coordination (JA) may contribute to the effective creation of CG during peer dialogue.

Moreover, little is known about the cojoined contribution of these variables—each interlocutor’s individual’s motor functioning together with the partners’ CG and JA—for successful peer communication among children and adolescents with autism spectrum disorder (ASD), who possess major challenges in peer interaction as a defining criterion (American Psychiatric Association, 2022). Therefore, this study aimed to empirically explore such language-motor links for this highly relevant population. Autism is characterized by challenges in social communication and restricted behaviors (*DSM-V-TR*, American Psychiatric Association, 2022) as well as by documented wide-ranging motor and motor coordination challenges (Bhat, 2020, 2021; Reynolds et al., 2022; Wang et al., 2022).

### Common Ground: Characteristics and Development in TD and ASD

CG refers to the shared meaning constructed by two interlocutors during a conversation, which is built upon their use of various speech acts such as an affirmation to an initiation, question asking, and correction of misunderstandings (Clark & Krych, 2004; Clark & Wilkes-Gibbs, 1986). Inasmuch as CG involves establishing shared knowledge and mutual understanding between speakers, its creation through conversational grounding makes everyday interactions more efficient. Difficulties in CG can result in lengthy, exhausting conversations that demand substantial effort to correct communication breakdowns (Clark & Wilkes-Gibbs, 1986; Okabayashi, 2017; Schuh et al., 2016).

Creating CG between two interlocutors necessitates the coordination of language, cognitive, and motor skills, and it involves recalling memory, delaying information, responding to gestures, and grasping the conversational context (Nilsen & Graham, 2009). In TD children, CG gradually evolves from early childhood (age 5 years) to preadolescence (age of 12 years) when children can initiate conversation corrections, offer clarifications, provide descriptions or gestures, and consider their partner’s perspective when facing gaps in their shared knowledge (Khu et al., 2020; Nadig & Sedivy, 2002).

Referential communication tasks are common procedures for CG evaluation (Abbot-Smith, et al., 2020; Bovet et al., 2024; Malkin et al. 2018a; Nilsen & Graham, 2009). They involve information exchanges where one speaker describes an unseen object, process, or image to another while relying on the ability to maintain and update CG representations with the partner (Schuh et al., 2016). Clark and Wilkes-Gibbs’s (1986) well-accepted referential communication task instructs pairs of TD adults to convey information (hidden from one partner) on geometric configurations’ arrangement, using tangram cards over six turns. Findings showed that neurotypical adults exhibited the benefits of CG creation over the course of this referential task by using the shortening effect, which reduces partners’ number of words and time needed to communicate and share knowledge effectively (Clark & Krych, 2004).

In autistic individuals, studies using various referential methods (such as tangram cards, computer games, and board games) have consistently shown less efficient CG creation compared to TD peers (Eigsti et al., 2011; Fukumura, 2016; Malkin et al., 2018b). For instance, in Nadig et al.’s (2009) referential communication task involving the creation of shared information and a guessing game, one participant was asked to give the partner a clue about a “secret” object hidden from the partner’s view. Autistic children and adolescents (ages 9–14 years) were found to use fewer informative terms and to include more unnecessary details when identifying objects, compared to their TD peers.

Referential communication shortening tasks were found to correlate with age and symptom severity in ASD; thus, older participants with less severe autism traits exhibited better referential shortening (De Marchena & Eigsti, 2016), calling for further investigation of the differences between ASD and TD youngsters’ developmental CG trajectories. Indeed, in the few available empirical studies on age trajectories for CG development in ASD, its creation was found to improve with age, with differences observed between younger (school-age) and older (adolescent) groups (Arnold et al., 2009; Fukumura, 2016). In Arnold et al.’s (2009) study, when describing a cartoon scenario to an adult partner, autistic children ages 9–12 years produced fewer references and used significantly more over-informative language compared to their TD age-mates; however, these group differences were not present in adolescents ages 13–17 years while communicating with an adult. Fukumura (2016) reported a developmental trend for CG among autistic participants, where younger children (6–10 years) tended to provide more unnecessary or missing information during referential tasks compared to older ASD participants (11–16 years) and to TD participants of all ages (6–16 years). This indicated that understanding of CG with an adult partner evolves along development for individuals with ASD. Other research on adults found that individuals with autism can establish shared knowledge but tend to do so more slowly and less efficiently than TD adults (Heasman & Gillespie, 2019; Nadig et al., 2015; Wadge et al., 2019).

In sum, research to date is quite limited on the process of CG creation during referential tasks and its development with age in both autistic and non-autistic populations (Arnold et al., 2009; Fukumura, 2016). Moreover, the available CG research in both groups has mainly focused on child–adult or adult-adult communication rather than children’s peer-to-peer interactions, which pose greater social demands especially for individuals with autism because adults tend to help mediate the interaction (De Marchena & Eigst, 2016). Thus, the performance of CG in autism during peer-to-peer interaction may be different and more challenging from the formation of CG with an adult as examined in most previous studies. Furthermore, prior research on CG growth during children’s peer-to-peer tasks and its developmental trajectory in ASD and TD has not yet sufficiently examined CG’s possible links with motor skills and JA synchronization in the context of the dyadic peer interaction. Overall, our study's novel contribution is its examination of the process of CG creation over 6 turns, in the context of peer-to-peer interaction, in children and adolescents, comparing ASD and TD, as well as the understanding of language-CG links with motor skills and synchronization.

### Motor Ability and Joint Action in TD and ASD

Although motor challenges are not among the formal criteria for diagnosing autism, recent reviews have underscored this population’s profound difficulties in gross-motor coordination, locomotor skills, fine-motor coordination, gait and posture, imitation, pantomime, and motor planning throughout the developmental stages from preschool to adulthood (Bhat, 2020, 2021; Licari et al., 2020; Zampella et al., 2020). Motor irregularities are already evidenced during infancy and toddlerhood by autistic individuals’ delayed acquisition of motor milestones. Later, despite a slight reduction in the motor dysfunction of autistic individuals with age (Fournier et al., 2010), motor challenges remain apparent in adolescence (Estrugo et al., 2023) and adulthood (Linke et al., 2020).

Beyond the individual’s own fundamental motor abilities, the child’s capacity to coordinate movements in time and space with a partner to achieve a shared goal—JA synchronization with a peer—underlies every human interaction (Knoblich & Sebanz, 2008). JA is built upon the individual’s ability to track, interpret, and predict their partner’s social-communicational and motoric intentions (Azaad & Sebanz, 2023). These capabilities are challenging for autistic individuals, as seen in several recent reviews that demonstrated significantly better JA performance in TD compared to autistic age-mates, alongside delayed maturation of JA in ASD (Cerullo et al., 2021; McNaughton & Redcay, 2020). A recent study supported this TD group advantage over the ASD group for dyadic motor coordination among children ages 6–16 years while performing JA tasks like shared walking and playing imaginary soccer with a same-age peer partner (Bar Yehuda & Bauminger-Zviely, 2022).

### Language-Movement Links: Common Ground, Motor Skills, and Joint Action

As noted, effective communication in a conversation activates both motor and language coordination skills, which contribute to the conversation’s flow and reciprocity. For example, the interplay between lexical, postural, and gestural mental representations is important to reach shared understanding between interlocuters (Galantucci & Sebanz, 2009; Garrod & Pickering, 2009). Each partner in the conversation coordinates talking with their own bodily movements but also coordinates their talk and motor actions with the other person’s timing, rhythm, and spatial orientation (Mondada, 2016). During a conversation, partners often employ joint motor and language actions, and they may reach a higher level of coordination by imitating and synchronizing their voice volume, gestures, gaze direction, and body posture (Okabayashi, 2017; Wynn et al., 2018). This motor-language coordination may impact partners' ability to cooperate, establish social relationships, and empathize with each other (Garrod & Pickering, 2009; Pickering & Garrod, 2004).

CG is a pragmatic ability, which denotes the communicative use of language for an efficient interaction. Pragmatic language encompasses a diverse range of abilities, such as mastering reciprocal conversational skills like turn-taking and adapting word choice according to different conversational contexts (De Marchena & Eigsti, 2016). Deficiencies and delays in both motor and language-pragmatic functioning were documented for autism. For instance, caregivers of almost 80% of autistic toddlers (mean age: 18.7 months) raised concerns about motor and language development before their toddler’s diagnosis (Herkert et al., 2023). Likewise, caregivers reported that their autistic toddlers (mean age: 2.3 years) presented significantly delayed attainment of both motor and speech developmental milestones (Matson et al., 2013). At later ages, the conversations of children and adolescents with autism (ages 9–16 years) showed both reduced movement (e.g., tempo similarity, coordination, simultaneous movement, posture congruence) as well as lower verbal synchrony (e.g., balanced and reciprocal conversations, coordinated pauses in speech) compared to same-age TD children (Zampella et al., 2020).

Moreover, these two skill areas have been linked in some prior research. Caregiver-reported motor and language-pragmatical performance by autistic children and adolescents (2–17 years) highlighted a positive association between fine-motor performance and expressive and receptive language skills (Mody et al., 2017). Also, autistic children and adolescents (22 months to 16 years) who participated in motor-based interventions demonstrated improvements in language proficiency skills such as social responsivity, word production, and linguistic flexibility (Odeh et al., 2022). Though the link between motor and pragmatic mechanisms in autism may postulate an interesting perspective on their peer-interaction functioning, the nature of the connection between JA and CG and the contribution of motor functioning to pragmatic coordination has not yet been sufficiently explored.

### Current Study

Using growth curve analyses for both ASD and TD groups, this study examined peer dyads’ process of CG creation evolving over a six-turn task, as reflected in dyads’ reduction of the number of words and time duration needed to successfully communicate referential information. We also investigated group differences (ASD, TD) and age differences (early childhood, preadolescence, adolescence) in dyads’ CG creation outcomes—by calculating word count and turn duration at the end of the task (the mean of the fifth and sixth turns) while controlling for baseline CG level (mean of the first and second turns). Lastly, we examined CG’s links with individual motor skills and dyadic JA synchronization and examined these motor variables’ contribution to CG creation. Although JA includes activation of both dyadic mirroring activities (imitating another’s movement) and dyadic complementing activities (reacting to and continuing another’s movement), we opted to use only complementary JA tasks for this study because both CG tasks and complementing JA tasks involve one partner’s action (describing a card, kicking an imaginary soccer ball) that elicits a complementary reaction (placing a card, catching an imaginary ball) from the partner.

Regarding growth in the efficacy of CG creation, we hypothesized a decrease from the first turn to the final turn in peer dyads’ number of words and turn duration in both research groups. We also expected better CG creation outcomes (at Turns 5–6, controlling for baseline) in the TD group than the ASD group, marked by shorter duration and reduced word count (e.g., De Marchena & Eigsti, 2016; Fukumura, 2016; Malkin et al., 2018a). Furthermore, we expected that both study groups would demonstrate better CG performance with age (e.g., Dahlgren & Sandberg, 2008; De Marchena & Eigsti, 2016; Fukumura, 2016).

In addition, expecting that individual motor skills and dyadic motor coordination ability would enhance dyads’ pragmatic coordination during social interactions, we hypothesized that child's better individual motor abilities and JA skills would correlate with lower CG scores (indicating lower word count and shorter turn duration, namely better CG efficiency), in both study groups, and we expected that both motor skills and JA would significantly positively contribute to the CG creation process (Zampella et al., 2020). Finally, considering that CG’s development is influenced by age and attains maturity during adolescence (De Marchena & Eigsti, 2016), we hypothesized that the link between JA and CG would be lower as a function of age increase.

## Method

### Participants

Participants were 148 children including 64 TD participants (16 girls, 50 boys) and 84 with ASD (14 girls, 70 boys), comprising three age levels: early childhood (6.0 to 8.5 years), preadolescence (> 8.5 to 12.0 years), and adolescence (> 12.0 to 16.0 years). As seen in Table 1, the ASD and TD groups were matched by participant's IQ, age, sex, and mother’s education, representing socioeconomic level. Due to the fact that CG may develop in autism later than in TD, we extended participants’ age range (6–16 years) to be able to capture possible delay in CG maturation (Nilsen & Graham, 2009). We started with age 6 because a pilot study on 8 TD children ages 5–16 years found that the 6-turn tangram card model was too long and difficult to perform before this age.

**Table 1:** Participant characteristics and clinical phenotyping

Background measures		Autism (ASD) group (*n* = 84)			Typically developing (TD) group (*n* = 64)			Statistical test
		Early childhood *n* = 22	Pre-adolescence *n* = 30	Adolescence *n* = 32	Early Childhood *n* = 22	Pre-Adolescence *n* = 20	Adolescence *n* = 22	
Chronological Age (in months)	*M*	91.86	120.77	169.91	86.77	127.50	172.09	*F*(142) = 2.10
	*SD*	(8.53)	(12.94)	(16.89)	(9.76)	(11.43)	(19.16)	
Mother’s education^a^	*M*	5.18	4.72	5.22	5.68	5.65	5.82	*F*(141) = 1.22
	*SD*	1.14	1.25	1.31	57	1.14	73	
*Sex*								
Male		20 (91%)	26 (87%)	24 (75%)	18 (82%)	16 (80%)	14 (64%)	${x}^{2}\left(1\right)$=1.56
Female		2 (9%)	4 (13%)	8 (25%)	4 (18%)	4 (20%)	8 (36%)	
Cognitive ability (IQ)	*M*	102.27	109.17	100.94	117.95	108.25	116.82	*F*(142) = 1.41
	*SD*	(28.15)	(32.27)	(32.46)	(30.54)	(20.67)	(15.24)	
ASD severity (ADOS-2)	*M* *SD*	7.50	6.70	6.41	–	–	–	*F*_ASD_(81) = 3.70,* p* < .05 Early childhood > Adolescence
	*M*	1.44	1.62	1.34				

ADOS-2: Autism Diagnosis Observation Schedule–2nd ed

^a^Mother’s education: 1 = elementary, 2 = high-school, 3 = matriculation, 4 = non-academic higher education, 5 = BA, 6 = MA, 7 = PhD

Inclusion criteria for the ASD group were: (1) Confirmation of prior clinical ASD diagnosis using the Autism Diagnosis Observation Schedule (ADOS-2, Lord et al., 2012) by the third author. (2) An IQ score of 70 + on the WISC-IV-HEB (Wechsler, 2010) to denote cognitively able children, using the full test for the ASD group and a mean IQ score (MeanVM) deriving from two subtests for the TD group: vocabulary (V) and matrices (M). Previous research indicated that these two subtests reliably reflected TD participants’ cognitive ability in verbal (V) and perception (M) domains (Brezis et al., 2017; Trevisan et al., 2021). Groups were matched according to the MeanVM.

#### Recruitment and Matching

This sample derived from a larger research project examining the role of motor functioning in social interaction. After receiving permission from the university faculty’s ethics committee, autistic and neurotypical participants were recruited to that project via social networks in Israel. All permissions and informed consent were obtained from parents. Over 3 years, 212 children (128 ASD and 84 TD) were recruited and then underwent matching procedures to generate dyads matched by (a) chronological age, with no more than 12 months between partners, (b) sex, and (c) IQ score (MeanVM) no more than one standard deviation between partners. This led us to exclude 34 children (14 ASD and 20 TD) who could not be matched with an appropriate peer. In addition, 30 ASD children were excluded because they did not meet the cognitive inclusion conditions (IQ < 70) or did not cooperate in the preliminary tests. As seen in Table 1, our final number of participants comprised 32 TD dyads (*n* = 64) and 42 ASD dyads (*n* = 84) at three age levels.

### Measures

Participants completed the 6-turn CG task, 8-part motor skills measure, and 2-part complementary JA task.

#### Common Ground Task

Using a referential communication paradigm, the dyadic CG task designed by Clark and Wilkes-Gibbs (1986) involved two peer partners communicating a tangram card model under confidential knowledge conditions, namely where the arrangement was known to the facilitating partner but concealed from the operating partner. As seen in the Appendix, the facilitator was instructed to describe the observable tangram shapes and their arrangement to the operator, who did not see the shapes but had to replicate the pattern by placing cards on the board based on the facilitator’s explanation. Dyads repeated this semi-structured CG task six times while exchanging roles (with three chances as facilitators and three as operators), utilizing the same 10 tangram shapes arranged in different patterns each turn. The youngest group (6–8 years) used only 6 tangram cards, with results standardized for comparison to the middle and older groups who used all 10 cards (standardization formula: multiply by 10 and divide by 6). The CG referential task was filmed with three cameras from three shooting angles (center, left, and right) to examine participants’ full responses.

For coding of the CG task, accurate performance was defined as a full match between the operator’s and facilitator’s card patterns. To assess growth in the efficiency of the referential CG creation process developing from Turn 1 to Turn 6, two main measures were coded at each turn: word number and turn duration (Clark & Krych, 2004; Clark & Wilkes-Gibbs, 1986). The *number of words* spoken by the facilitator and the operator during each of the six turns was counted using transcripts of the videos by the first author, a senior speech therapist, and was verified by a word counting application (http://www.yo-yoo.co.il/tools/wordcounter/). Each of the six turns’ *duration time* was measured from starting point (the facilitator’s first instruction) to endpoint (the operator’s placement of the last card on the board) and was verified by comparison to time durations using a media player application (Windows Media Player®). Shorter turn duration and lower word count from Turn 1 to Turn 6 reflected more efficient CG performance.

#### Child’s Motor Abilities

Each individual child’s gross and fine motor abilities were evaluated using the 8-item Individual Motor Observation Scale (IMOS), created for this research project by Bauminger-Zviely et al. (2017) in collaboration with movement and occupational therapy professionals. The 8 IMOS tasks (Cronbach α = 0.86) examined *upper-body gross motor skills* (2 tasks: bouncing a ball, throwing and catching a ball toward/from a wall) and *lower-body gross motor skills* (3 tasks: skipping, hopping on one leg, heel-to-toe walking) and *fine motor skills* (3 tasks: cutting a straight and a curved line with a pair of scissors, nailing three nails in a row with a hammer). Two special education experts separately coded children’s videotaped IMOS performance. Each coder first completed 25% of the videos, yielding 94% agreement between coders for gross motor and 93% for fine motor skills, which enabled continued coding of the remaining videos. We utilized the total IMOS motor score in this study based on preliminary analysis revealing its strong correlations with the IMOS gross (*r* = 0.98, *p* = 0.000) and fine (*r* = 0.75, *p* = 0.000) motor components (see Estrugo et al., 2023).

#### Complementary Dyadic Joint Action

Using Bar Yehuda and Bauminger-Zviely’s (2022) two complementing JA measures, the fixed peer dyads engaged in a *corridor task* (walking towards the partner and adjusting body position to pass each other within an imaginary narrow corridor) and a *soccer task* (kicking and catching an imaginary soccer ball). Coding assessed participants’ coordinated movement score: their co-occurring body positioning movement (corridor) and coordinated kick-and-catch movement (soccer) that complemented their partner’s movements. This score was calculated as the ratio of the coordinated movement's duration or frequency performed simultaneously by both participants to the total number of movements performed by each participant (see Bar Yehuda & Bauminger-Zviely, 2022 for details on JA tasks and coding procedures).

### Procedure

For the current study, two sessions were conducted at our autism research laboratory. The first session involved assessing the ASD diagnosis (for the ASD group) and cognitive abilities (for both groups). The second session, administered by our research team (third and fourth authors), focused on assessing CG and JA tasks during peer interactions and the IMOS, in counterbalanced order.

### Data Analysis

#### Common Ground Performance Accuracy

To assess accuracy of CG performance, percentages were calculated for each study group’s mean number of tangram cards placed correctly during Turns 5 and 6.

#### Common Ground Growth Curve

To determine growth trends in CG creation along the six turns, we applied the Individual Growth Curve Model (Hoffman, 2015; Walters & Hoffman, 2017). This procedure estimates development parameters, that is, estimating an individual’s intercept and slope as latent variables as well as an overall mean intercept and slope (Geiser, 2013). In this study, the individual referred to a single dyad of two jointly practicing partners along six turns, while estimating trends for the two outcome measures: word count and turn duration. We used the Mplus V.8.3 (Muthén & Muthén, 2018) statistical package to estimate growth parameters. In practice, we assessed an intercept (the level at turn of entry) and a slope (a linear change from one turn to another) for each pair (dyad) of participants, namely, the individual’s growth parameters. These individuals’ parameters were used as outcomes to estimate the group (TD versus ASD) effect on CG development, that is, to assess possible group differences in the growth parameters. The advantage of the growth modeling approach is its provision of trend parameters (e.g., intercepts and slopes), beyond mere differences. In addition, the growth model includes measurement errors at each turn.

#### Group and Age Differences in Common Ground

To evaluate Group, Age, and Group × Age effects on CG creation outcomes (mean of Turns 5–6 for word count and turn duration), we used analysis of covariance (ANCOVA) while controlling for the role played by participants' baseline CG level (means at Turns 1–2). The source of the Group × Age interaction in all analyses was determined using post hoc pairwise comparisons adjusted by Bonferroni’s (1936) correction, subject to the *p* < 0.05 rejection criterion.

#### Correlational Analyses

A series of partial correlation tests was conducted for the two CG outcome variables of word count and turn duration at Turns 5–6 (while controlling for baseline variables at Turns 1–2), to assess their links with motor skills (IMOS) and with complementary JA coordination.

#### Moderated Mediation Model for Common Ground

To further understand how the relations between individual motor skills, JA, and age may contribute to CG creation, we employed the SPSS add-on PROCESS macro moderated mediation model 87 (Hayes, 2018). This procedure allows for the examination of direct and indirect effects of the predictor X (study group) on the dependent variable Y (CG) through the mediation of motor skills and JA, with age as moderator (e.g., Ahuja et al., 2022). Significance of the mediation effect was estimated using 95% confidence interval (CI), calculated based on bootstrapping of 10,000 samples.

## Results

### Preliminary Analyses: Common Ground Performance Accuracy

Participants revealed very high accuracy rates in placing the CG tangrams correctly at Turns 5–6. Of the TD dyads, 97% achieved a full match between facilitator and operator toward the end of the CG task, compared to 95% in the ASD group, yielding non-significant group differences. Due to these very low failure percentages, accuracy was not entered into our analyses.

### Growth Curve in Common Ground

#### Word Count

Estimation of growth parameters for the number of words spoken across the six turns of the CG task revealed that individual dyads (the observational unit of this analysis) showed a starting level (mean intercept) of 96.297, *p* < 0.001 (95% CI [76.12, 136.68]) and a unit reduction (mean slope) of −9.714, *p* = 0.001 (95% CI [−19.53, −5.81]). We noted that the overall drop in word count was steeper in the first part of the task (Turns 1, 2, 3) and showed a more moderate slope in the second part (Turns 4, 5, 6). Thus, we adopted the piecewise approach to fit different slope parameters for each set of time points. For the total sample, as expected, the slope for the beginning of the task was significantly steeper (Slope_1–3_ = −37.618, *p* < 0.001, 95% CI [−49.93, −21.01]) than the slope for second part of the task (Slope_4–6_ = −4.916, *p* < 0.001, 95% CI [−9.39, −2.56]. On average, the drop in word count was around 40 words per additional turn along the first three turns, compared to an average drop of only 5 words per additional turn along the last three turns.

**Fig. 1:**
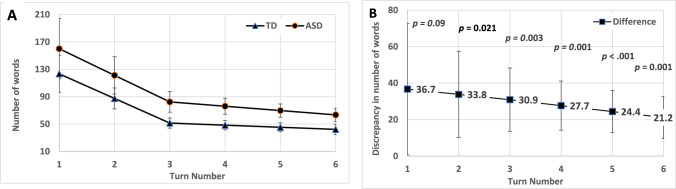
Piecewise Plot of Word Count Trends by **A** Group and **B** Group Discrepancies (ASD Minus TD Means) at Each Turn in Common Ground Task. *Note.* Vertical lines are error bars for 95% CI; *p v*alue for significant difference

To examine possible distinct developmental curves for the two study groups, we added group (ASD, TD) as an additional effect on the random intercept and the two random slopes. This model resulted in a slight discrepancy between the two intercepts (*d* = 36.74, *p* = 0.090, 95% CI [0.67, 85.26], where *d* stands for parameter discrepancy of ASD less TD. However, group slopes did not show a significant discrepancy (*d*1 = −2.91*, p* = *0.7*37*,* 95% CI [−0.87, 12.67]; *d*2 = −3.26*, p* = 0.285; 95% CI [−10.27, 2.15]). When comparing this discrepancy at each time point (i.e., turn), the ASD versus TD group difference became significant from Turn 2 onward. Figure 1A illustrates word count trends by group, and Fig. 1B presents the group discrepancy in predicted values (ASD minus TD means) for word count at each turn. As seen in Fig. 1B, although the largest discrepancy in word count between the ASD group (*M* = 160.1 words, SE = 27.1) and the TD group (*M* = 123.4 words, SE = 44.0) emerged at the starting point (Turn 1), it did not reach significance due to large within-group variance (*d* = 36.74, *p* = 0.097, 95% CI [−1.28, 92.44]). No group effect emerged on either of the two slopes, although calculated discrepancies were found to be significant (*p* < 0.05) at each turn except Turn 1 (*p* = 0.09). To summarize, although the largest mean group difference emerged at the first turn, it was not significant due to high within-group variation. Slopes of the first to third turns and the fourth to sixth turns did not vary by group; but the next turns (second to sixth) showed a significant yet diminishing group difference favoring the conciseness (lower word count) of the TD group over the ASD group.

#### Turn Duration

**Fig. 2:**
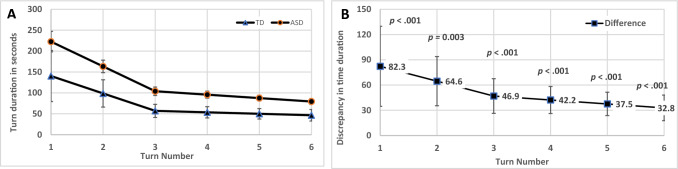
Piecewise Plot of Turn Duration Trends by **A** Group and **B** Group Discrepancies (ASD Minus TD Means) at Each Turn in Common Ground Task. *Note.* Vertical lines are error bars for 95% CI; *p* value for significant difference

Similarly, we analyzed the turn duration (in seconds) controlled by the group effect (TD vs. ASD). As seen on Fig. 2A, at the beginning of the CG task (Turns 1–3), the TD group showed a steep reduction in turn duration, but then for Turns 4–5 the trend remained relatively flat (Intercept = 140.38*, p* < 0.001, 95% CI [114.07, 177.99]; Slope_1–3_ = −41.67, *p* < *0.0*01, 95% CI [−57.53, −31.30]; Slope_4–6_ = −3.50, *p* = 0.063, 95% CI [−7.99, −0.48]). In the ASD group, duration was longer at the beginning but dropped even faster and continued along the next turns (Intercept = 222.63, *p* < 0.001, 95% CI [161.02, 276.66]; Slope_1–3_ = −59.32, *p* < 0.001, 95% CI [−86.25, −31.31]; Slope_4–6_ = −8.20, *p* = *0.0*02, 95% CI [−14.21, −3.49]). Figure 2A illustrates these trends for the two groups, showing the same pattern of group differences in turn duration as emerged for word count (ASD effect on growth parameters: Intercept b = 82.25, *p* = 0.003, 95% CI [34.65, 138.10]; Slope_1–3_ b = −17.65, *p* = 0.148; Slope_4–6_: b = −4.71, *p* = 0.191), namely, ASD was found to differ in the intercept, but not in the slopes. Figure 2B shows that the predicted discrepancies between groups remained significant but diminished in value over time.

#### Overall Growth Trends

To summarize, results demonstrated a similar overall trend in CG creation for both study groups, namely a reduction in dyads’ number of words and turn duration from Turns 1 to 6, despite preliminary group differences favoring the TD group at the starting point (Turn 1) that reached significance for turn duration and neared significance for word count. Although discrepancies between the groups for word count (Fig. 1B) and turn duration (Fig. 2B) diminished over time, the ASD group lagged behind the TD group in their efficiency of CG creation.

### Group and Age Differences in Common Ground Efficacy Outcomes

The 2 (group) × 3 (age) ANCOVA for word count and turn duration outcomes (means at Turns 5–6, controlling for mean baseline CG at Turns 1–2) yielded significant group and age effects and a nonsignificant group X age interaction. As seen in Table 2, outcomes showed that the TD group used fewer words and completed the task more quickly than the ASD group toward the end of the CG task. Improvement with age also emerged for both outcome measures. Regarding word count, adolescents used significantly fewer words than preadolescents, who in turn used significantly fewer words than the youngest group. Regarding duration, the adolescent group needed significantly less time to complete the task, compared to both the preadolescent and early childhood groups. The non-significant group by age interaction suggested that improvement with age emerged for both study groups.

**Table 2:** Means, standard deviations, F, and partial eta squared values for common ground efficacy outcomes: word count and turn duration by group and age

Outcomes		Autism (ASD) group *n* = 84			Typically developing (TD) group *n* = 64			Group *F* (1,141), η_p_^2^	Age *F* (2,141), η_p_^2^
		Early childhood *n* = 22	Pre-adolescence *n* = 30	Adolescence *n* = 32	Early childhood *n* = 22	Pre-adolescence *n* = 20	Adolescence *n* = 22		
Word count	*M*	80.38	64.33	61.06	54.05	48.17	30.77	18.72^***^ .12	15.75^***^, .18 Early childhood > Preadolescence > Adolescence
	*SD*	(36.84)	(25.91)	(41.50)	(22.78)	(18.65)	(16.88)		
Turn duration	*M*	112.05	79.06	62.34	58.23	57.02	30.45	19.05^***^ .12	12.49^***^, .15 Early childhood > Preadolescence > Adolescence
	*SD*	(50.65)	(50.25)	(34.57)	(24.15)	(27.49)	(6.57)		

Outcomes refer to words and duration means at Turns 5–6, controlling for means at baseline (Turns 1–2)

***p < .001

### Common Ground Correlations with Motor Skills and Joint Action

Partial correlation tests yielded significant negative correlations between participants’ motor skills (IMOS) and CG efficacy outcome variables (at Turns 5–6, while controlling for baseline CG at Turns 1–2) for both word count (TD: *r* = −0.55, *p* < 0.001; ASD: *r* = −0.19, *p* = *0.0*42) and turn duration (TD:* r* = −0.36, *p* = 0.002; ASD:* r* = −0.27, *p* = 0.007). This indicated that higher individual motor abilities were significantly linked with lower word count and shorter turn duration in both groups and provided a justification for the further modeling approach. Likewise, significant negative correlations emerged between complementary JA scores and both CG outcomes: word count (TD: *r* = −0.48, *p* < *0.0*01; ASD: *r* = −0.34, *p* = *0.0*01) and turn duration (TD: *r* = −0.32, *p* = 0.006; ASD:* r* = −0.28, *p* = 0.005), where correlations above 0.30 were considered meaningful to some extent, regardless of the null hypothesis significance test results (0.30 < *r* < 0.50 low; 0.50 < *r* < 0.70 moderate; Hinkle et al., 1988). This indicated that dyads’ higher JA synchronization was linked with fewer words and faster duration in both groups. Overall, higher motor abilities and socio-motor synchronization were both linked with more efficient CG performance. Importantly, the TD group showed stronger correlations in comparison to the ASD group, which suggested that TD group was relatively more homogeneous in comparison to the ASD group. In other words, the TD group’s empirical correlations coincide better with the theoretical model than the ASD group’s.

### Moderated Mediation for Common Ground

**Fig. 3:**
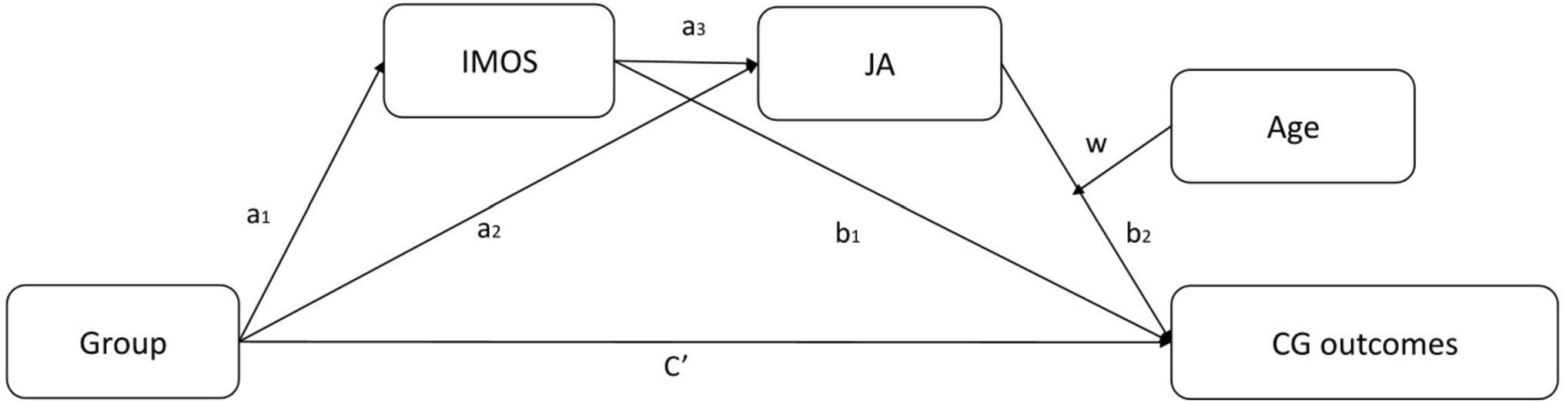
Indirect Relationship Between Group (ASD, TD) and Individual Motor Functioning (IMOS) Through Dyadic Joint Action (JA) to Common Ground (CG) Outcomes, With Age Level Moderating the Relationship (N = 148). *Note*. Moderated mediation model 87. The CG outcomes refer to word count and turn duration means at Turns 5–6, controlling for means at baseline (Turns 1–2). Indirect effect path 1. Group → IMOS → JA: (w)- CG: a1- a3-w|b2. Indirect effect path 2. Group → IMOS → CG: a1-b1. Indirect effect path 3. Group → JA: (w)- CG: a2-w|b2. Direct effect. Group → CG: c'

Table 3 and Fig. 3 present our moderated mediation analysis using the SPSS add-on PROCESS macro model 87 (Hayes, 2018) to test whether the indirect relationship between group (TD/ASD) and CG (words and duration at Turns 5–6, controlled by Turns 1–2) was mediated by IMOS and JA and was moderated by age level. A negative association emerged in the path between group and IMOS, indicating better IMOS in the TD group (which was coded 0, versus the ASD group coded 1). A positive association emerged between IMOS and JA, indicating that better individual motor abilities were linked with better dyadic synchronization. In turn, JA was negatively associated with CG, indicating that fewer words and shorter duration in CG outcomes were linked with higher complementary JA synchronization.

**Table 3:** Significant moderated mediation effects for conditional indirect relationship between group (ASD, TD) and common ground outcomes via individual motor functioning (IMOS) and dyadic joint action (JA) by age level (Early, Preadolescent, Adolescent)

Common ground outcome: number of words						
Predicted variable	Predictor	*b*	*SE*	*t*	*p*	95% CI
IMOS	Group	−9.37	1.11	−8.42	< .001	[−11.57 to −7.18]
JA	Group	−4.01	3.19	−1.27	= .21	[−10.37 to 2.25]
	IMOS	.83	.19	4.24	< .001	[.44 to 1.21]
Common Ground	Group	16.19	5.59	2.90	= .004	[5.14 to 27.24]
	IMOS	.17	.38	.45	= .65	[−.57 to .91]
	JA	−1.23	.43	−2.83	= .005	[−2.08 to −.37]
	Age	−.79	.27	−2.88	= .005	[−1.34 to −.25]
	JA × Age	.008	.004	2.04	= .043	[.0002 to .02]
Conditional indirect effects	For 90 mo	−.54	.15	−.3.58	< .001	[−.83 to−.24]
	For 129 mo	−.24	.15	−1.66	= .097	[−.53 to .05]
	For 172 mo	.08	.26	.30	= .761	[−.44 to .59]
*Common ground Outcome: Turn duration*						
IMOS	Group	−8.35	1.13	−7.34	< .001	[−10.59 to −6.11]
JA	Group	−4.18	3.20	−1.31	= .19	[−10.51 to 2.15]
	IMOS	.77	.20	3.86	< .001	[.38 to 1.17]
Common Ground	Group	21.86	8.52	2.56	= .011	[5.01 to 38.71]
	IMOS	−.29	.57	−.51	= .61	[−1.43 to .83]
	JA	−1.68	.64	−2.64	= .009	[−2.95 to −.42]
	Age	−1.07	.40	−2.71	= .008	[−1.86 to −.29]
	JA × Age	.012	.005	2.13	= .035	[.01 to .02]
Conditional indirect effects	For 90 mo	−.64	.23	−2.81	= .006	[−1.01 to −.18]
	For 129 mo	−.19	.22	−.89	= .37	[−.62 to .23]
	For 172 mo	.30	.38	.79	= .43	[−.45 to 1.04]

Moreover, age moderated the association between JA and CG outcomes; that is, the JA—CG association was found to vary by age. To determine this variation, we looked at the JA—CG association conditioned by different ages. This decomposition revealed that the negative association between JA and CG was weaker at older ages and became non-significant above age 125 months for word count and above 112 months for turn duration. In other words, the relationship between study group and CG outcomes was moderated by JA only for children younger than 10 years. For those younger children, better individual and dyadic JA motor skills increased the efficiency of CG.

## Discussion

This study investigated variations in CG creation among autistic and TD peer partners across three developmental stages, as well as the contribution of their individual motor skills and dyadic motor coordination (JA) abilities to partners’ creation of language coordination (CG). The study design was unique in observing reciprocal dialogue and movement occurring between two peers, which are more challenging than in scaffolded interactions with adults and may not naturally transfer from child–adult into peer settings, specifically for youngsters with autism (e.g., Dekker et al., 2016). Our focus on dyads’ joint construction of referential CG and the role of motor coordination in their language coordination may propose novel understanding of movement–pragmatic links, which may open new therapeutic channels.

### Group and Age Differences in Common Ground

One of the most intriguing findings of this study was that, like their TD age-mates, autistic children reached high CG accuracy levels indicating that they were able to construct CG over six repeated rounds of the semi-structured tangram task, by reducing the number of words and time they required to describe each shape to their peer partner and successfully communicate its target position on the board. This improvement reflects the participants’ growing capacity to incorporate and assimilate dynamic ongoing linguistic information that develops throughout interaction (Abbot‐Smith et al., 2020; Baixauli-Fortea et al., 2019; Brennan et al., 2010; Dahlgren et al., 2008; Matthews et al., 2018; Resches et al., 2007). Furthermore, the piecewise plotting showed that most of the improvement in establishing shared meaning between partners (faster solutions and shorter referential forms) seemed to occur during the first three turns (with the steeper slope showing this change). This may imply that even a shorter training task could be effective to generate CG and enhance pragmatic knowledge via future interventions.

Growth curves across the six turns showed diminishing differences between TD and ASD groups over time, suggesting autistic participants’ spontaneous learning process through peer interaction. Yet, despite their increasing efficiency in CG creation, the autism dyads continued to need more words and more time to communicate the tangrams across the task, compared to the TD dyads. This less efficient and more prolonged grounding that the autistic participants demonstrated during the CG creation process coincides with prior studies that examined CG in child–adult situations (e.g., Clark & Wilkes-Gibbs, 1986; de Marchena & Eigsti, 2016; Nadig et al., 2015). Their disadvantage can be understood in terms of autistic children’s multiplex social-cognitive-pragmatic challenges in areas essential for CG creation such as information processing and categorization, working memory, part-whole understanding, and theory-of-mind perspective taking (Malkin et al., 2018b; Matthews et al., 2018; Schuh et al., 2016).

The developmental trajectory found for dyads’ CG creation was also similar in the two study groups; hence, with age, peer partners used shorter referential forms (fewer words) and completed the task faster. These novel findings regarding CG improvement across development in ASD suggest a natural linguistic maturation and social skills expansion during peer interaction.

### Relations Between Individual and Dyadic Motor Abilities and Common Ground

As expected, negative correlations emerged between CG abilities with individual motor skills and with JA synchronization, in both study groups. Additionally, the finding that partners’ age was a moderating factor in JA’s relationship with CG creation suggests a more pronounced indirect link between group and CG in younger children (under age 10 years; 4 months for word count and 9 years; 3 months for turn duration). Possibly, CG reaches maturation during preadolescence; thus, involvement of the dyadic motor coordination (JA) system may be less necessary to the creation of language coordination (CG) at this age, in contrast to younger ages. This finding suggests that early interventions targeting individual and dyadic motor abilities before age 10 could amplify later pragmatic language maturation during adolescence in ASD.

The current study expanded on the role of age in the language-body link, thereby extending prior research indicating that motor, JA, and language abilities improve with age in autistic children. Szokolszky and Kékes Szabó (2019) reported potential improvements in motor functioning, specifically in motor apraxia, delicate motor control, and coordination during puberty in individuals with autism, although during childhood greater motor dysfunction may still be exhibited in comparison to TD counterparts. Bar Yehuda and Bauminger-Zviely (2022) showed improvement in JA performance with age in ASD; however, autistic children lagged behind their TD age-mates. Eigsti et al. (2011) described the improvement of pragmatic verbal abilities as crucial in adolescence, particularly for individuals with ASD, due to its contribution to the establishment of more effective CG for communication. Given these findings, there is a compelling case for early intervention to address motor and language challenges in children with autism, recognizing the potential positive impact on their developmental trajectories.

Therefore, this study may suggest that interventions aimed at improving motor functioning could enhance the pragmatic abilities of autistic children, especially below age 10, thus offering deeper understanding of language development in the context of ASD and peer interaction (Lappalainen, 2018).

The significant motor-language link found in the current study also supports recent research on child–adult rather than peer interactions, using various questionnaires (Hannant, 2018; Stevenson et al., 2017; Wu et al., 2021). Similarly to our study, these prior researchers showed a positive correlation between language abilities (both receptive and expressive) and motor abilities (such as balance and fine- and gross-motor skills) in both TD and ASD groups. Furthermore, a correlation emerged between language and JA motor coordination during conversation in children with ASD and TD (Delaherche et al., 2013; Szokolszky & Kékes Szabó, 2019). In line with these links, researchers have suggested integrating motor components into intervention programs to enhance language skills, as proposed by Lappalainen (2018) and Odeh et al. (2022).

### Study Limitations, Implications, and Directions for Future Research

Our study has several limitations. Despite its sample of 148 participants, a larger number could enable more complex data analyses, for example the inclusion of age in addition to the CG turns in our growth modeling analysis. In addition, this study resembled prior CG research in utilizing a semi-structured tangram-card task; however, CG creation should also be examined during spontaneous day-to-day social conversation. Also, although this study focused on the key aspects of peer partners’ CG creation (word number and duration), future researchers would do well to focus on qualitative differences in shape description (e.g., mistakes and corrections to meet listener’s needs) between autistic and TD children. Likewise, it is important to expand coding procedures to include other discourse components such as pauses' duration, shared gaze, bodily gestures, and facial expressions to obtain a deeper understanding of dyads’ CG creation process in both study groups.

In conclusion, this study's importance lies in its two main findings. First, despite their socio-cognitive and linguistic challenges, autistic children were found to exhibit spontaneous improvement in their linguistic-pragmatic CG skills during peer interaction. Notably, this capability was found to improve from early childhood to adolescence. The second major finding was that both investigated motor mechanisms—the child’s individual motor abilities and ability to synchronize motor actions with a peer—contributed more effectively to achieving CG at younger ages (around 10 years and below) for both groups. Future studies should delve deeper into exploring the potential impact of both motor mechanisms on the pragmatic challenges of autistic children. This can include areas such as coordinating speech rate, course, and rhythm as well as body language, the use of gestures through discourse, and coordination of facial expressions, both in and outside of CG.

## Supplementary Information

Below is the link to the electronic supplementary material.

Supplementary file1 (DOCX 664 kb)
