# Correction to: Movement Coordination’s Link with Common Ground During DyadicPeer Discourse in Typically Developing and Autistic Speakers

**DOI:** 10.1007/s10803-025-06793-0

**Published:** 2025-03-17

**Authors:** Einat Karin, Ronny Geva, Shahar Bar-Yehuda, Yael Estrugo, Nirit Bauminger-Zviely

**Affiliations:** 1 Faculty of Education, Bar-Ilan University, Ramat-Gan, 52900 Israel; 2 Department of Psychology, The Gonda Brain Research Center, Bar-Ilan University, Ramat-Gan, 52900 Israel

**Correction To: Journal of Autism and Developmental Disorders (2024)**

10.1007/s10803-024-06642-6

In this article, the author’s name Nirit Bauminger-Zviely was incorrectly written as Nirit Bauminger Zviley.

The original article has been corrected.

