# Supplementary material for: Movement Coordination’s Link with Common Ground During Dyadic Peer Discourse in Typically Developing and Autistic Speakers
Source: J Autism Dev Disord. 2024 Nov 21;56(4):1325–38. doi: 10.1007/s10803-024-06642-6 (PMC12987844; doi:10.1007/s10803-024-06642-6)

**Appendix**

***Tangram Card Paradigm***

In the top picture, the facilitator (on the left) describes to the operator where to put each card according to the model visible only to her. In the bottom picture, at the end of the turn, the participants compare the two models.


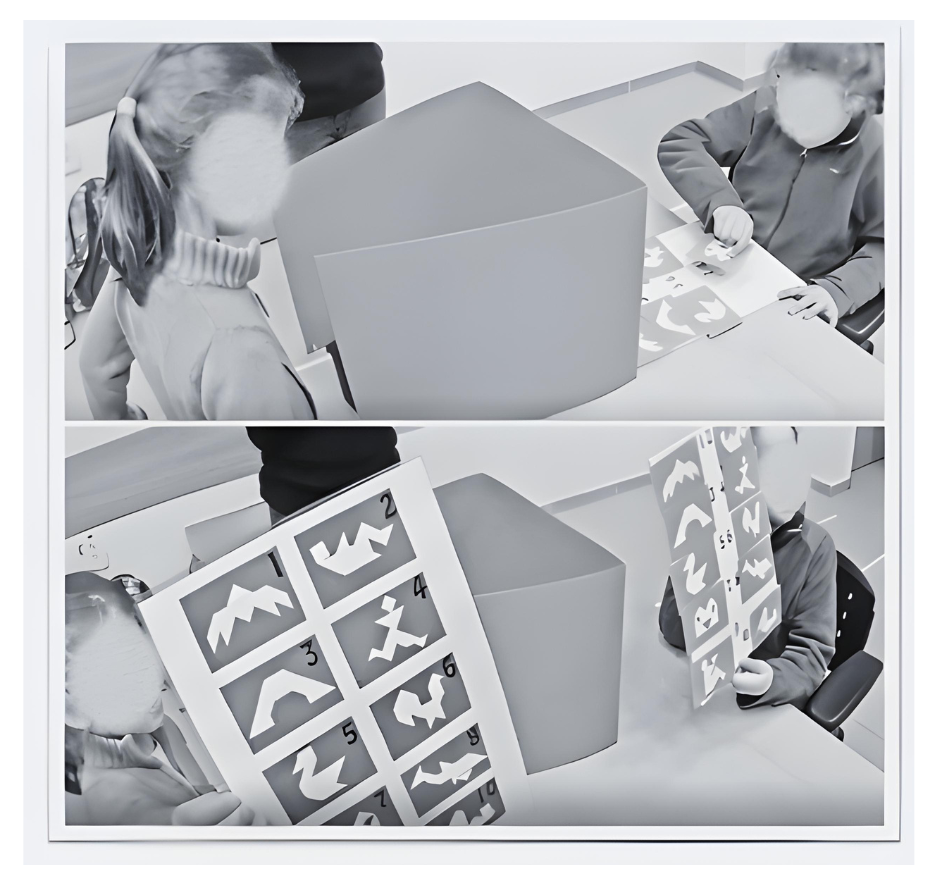

Supplement: Supplementary file 1 — Supplementary file1 (DOCX 664 kb) [file 10803_2024_6642_MOESM1_ESM.docx]
